# Adherence to a lifestyle intervention ‐ self‐efficacy as crux of the matter?Analysis of AgeWell.de as multi‐domain lifestyle intervention against cognitive decline

**DOI:** 10.1002/alz.086867

**Published:** 2025-01-09

**Authors:** Felix Georg Wittmann, Alexander Pabst, Andrea Zülke, Melanie Luppa, Birgitt Wiese, Wolfgang Hoffmann, Thomas Frese, Jochen Gensichen, Hans‐Helmut König, Hanna Kaduszkiewicz, Jochen René Thyrian, Steffi G. Riedel‐Heller

**Affiliations:** ^1^ Institute of Social Medicine, Occupational Health and Public Health (ISAP), Medical Faculty, University of Leipzig, Leipzig, Saxony Germany; ^2^ Institute for General Practice, Hannover Medical School, Hannover, Lower Saxony Germany; ^3^ Institut for Community Medicine, University Medicine, University of Greifswald, Greifswald Germany; ^4^ German Center for Neurodegenerative Diseases e.V. (DZNE), Site Rostock/Greifswald, Greifswald Germany; ^5^ Institute of General Practice and Family Medicine, Martin‐Luther‐University Halle‐Wittenberg, Halle, Saxony‐Anhalt Germany; ^6^ Klinikum der Ludwig‐Maximilians‐Universität München, München, Bavaria Germany; ^7^ University Medical Center Hamburg‐Eppendorf, Hamburg, Hamburg Germany; ^8^ University of Kiel, Kiel, Schleswig‐Holstein Germany; ^9^ Faculty V: School of Life Sciences, University of Siegen, Siegen, 57076 Germany; ^10^ German Center for Neurodegenerative Diseases (DZNE), Greifswald Germany; ^11^ Institute for Community Medicine / University of Greifswald, Greifswald Germany; ^12^ Institute of Social Medicine, Occupational Health and Public Health (ISAP), Medical Faculty, University of Leipzig, Leipzig Germany

## Abstract

**Background:**

Recent research on preventing cognitive decline has focused on lifestyle interventions, with first studies indicating cognitive benefits and suggesting a positive link between adherence to the interventions and their effectiveness. The purpose of this study was to analyse possible predictors of this very adherence to single components of a multi‐domain lifestyle intervention.

**Methods:**

A total of n = 317 participants of the intervention group were included, characterized with an age ≥60 (mean age 68.9) and an increased risk of dementia (CAIDE score of ≥9). Generalized linear regression models were used to regress four predictor blocks (sociodemographic factors, cognitive and psychosocial predictors, lifestyle factors and chronic conditions, all assessed at baseline) on the adherence to each of four components of AgeWell.de (nutritional counselling, enhancement of social and physical activity and cognitive training). Adherence was operationalised as mean score of seven time points at which a study nurse assessed the degree of goal achievement per component. The goals were set individually at beginning of the intervention.

**Results:**

Strengthening effects on adherence were found for higher education, unimpaired mental state and higher self‐efficacy. Increasing age, reporting depressive symptoms, smoking and partwise higher body mass index were instead negatively associated with adherence. No effect was found for chronic conditions.

**Conclusion:**

The study has identified both strengthening and mitigating predictors of adherence. This is relevant to future intervention designs aimed at enhancing adherence as a pivotal aspect for effectiveness. While age or education remain non‐modifiable, self‐efficacy emerge as a promising predictor. In light of optimizing the efficacy of forthcoming lifestyle interventions, a reasonable approach may involve the incorporation of strategies for enhancing self‐efficacy within the study design.

